# Progress in Surgery of the Stomach

**Published:** 1904-01-23

**Authors:** 


### PROGRESS IN SURGERY OF THE STOMACH.

*Concluded from page 277.*

Hour-glass Stomach and Trifid Stomach as a result of gastric ulcer are reported by Moynihan.[5] The symptoms of hour-glass stomach will naturally vary according to the position of the constriction in the stomach; if this lies near the cardiac orifice, the clinical picture will resemble that given by œsophageal obstruction low down; if near the pyloric orifice, the symptoms are those of dilated stomach. The following signs enable a diagnosis to be made in every case:—(1) If the stomach tube be passed, and the stomach washed out with a known quantity of fluid, the loss of a certain quantity will be observed when the return fluid is measured (Wölfler's "first sign"). (2) If the stomach be washed out until the fluid returns clear, a sudden rush of foul, evil-smelling fluid may occur; or if the stomach be washed clean, the tube withdrawn and passed again, in a few minutes several ounces of dirty offensive fluid may escape. The fluid has regurgitated through the connecting channel between the cardiac and pyloric pouches. (3) Paradoxical dilatation. If the stomach be palpated and a succussion splash obtained, the stomach tube passed, and the stomach apparently emptied, palpation will still elicit a distinct splashing sound. (4) Von Eiselsberg noted that on distending the stomach a bulging on the left side of the epigastrium was produced; after a few moments this gradually subsided and concomitantly there was a gradual filling up and bulging on the right side. (5) Von Eiselsberg mentions the bubbling, forcing, "sizzling" sound, which can be heard when the stethescope is applied over the stomach after distension with carbonic acid gas. (6) The abdomen is carefully examined and the stomach resonance percussed. A seidlitz powder in two halves is then administered. On percussing after about 20 or 30 seconds, an enormous increase in the resonance of the upper part of the stomach can be found, while the lower part remains unaltered. (7) Schmidt Monard and Eichorst have both seen a distinct sulcus between the two pouches, inflated with carbonic-acid gas. (8) When the stomach is filled with water and examined by gastro-diaphany the transillumination is seen only in the cardiac pouch, the pyloric pouch remains dark. (9) A deglutable rubber bag is passed and distended; the bulging caused thereby is limited to the cardiac pouch which lies to the left of the middle line. The following are the operations which may be practised for this condition. 1. Gastroplasty. 2. Gastro-gastrostomy or gastro-anastomosis. 3. Either of the foregoing, with gastro-enterostomy from the pyloric pouch in cases of dual stenosis. 4. Gastro-enterostomy from cardiac pouch when the pyloric pouch is so small that it can be ignored. 5. Gastro-enterostomy from both pouches. 6. Partial gastrectomy. To remedy the defect in the case of trifid stomach the first pouch was united to the second by the operation of gastro gastrostomy, an opening being made that would admit three fingers easily; the second pouch was united to the jejunum by a posterior gastro-enterostomy; and the constriction between the second and third pouches was dilated with the fingers.

[5] Brit. Med. Jour., June 13, 1903, p. 1366, and Lancet, Aug. 8, 1903, p. 388.

